# Global Percentage of Asymptomatic SARS-CoV-2 Infections Among the Tested Population and Individuals With Confirmed COVID-19 Diagnosis: A Systematic Review and Meta-analysis

**DOI:** 10.1001/jamanetworkopen.2021.37257

**Published:** 2021-12-14

**Authors:** Qiuyue Ma, Jue Liu, Qiao Liu, Liangyu Kang, Runqing Liu, Wenzhan Jing, Yu Wu, Min Liu

**Affiliations:** 1 Department of Epidemiology and Biostatistics, School of Public Health, Peking University, Beijing, China; 2 School of Health Humanities, Peking University, Beijing, China

## Abstract

**Importance:**

Asymptomatic infections are potential sources of transmission for COVID-19.

**Objective:**

To evaluate the percentage of asymptomatic infections among individuals undergoing testing (tested population) and those with confirmed COVID-19 (confirmed population).

**Data Sources:**

PubMed, EMBASE, and ScienceDirect were searched on February 4, 2021.

**Study Selection:**

Cross-sectional studies, cohort studies, case series studies, and case series on transmission reporting the number of asymptomatic infections among the tested and confirmed COVID-19 populations that were published in Chinese or English were included.

**Data Extraction and Synthesis:**

This meta-analysis was conducted following the Preferred Reporting Items for Systematic Reviews and Meta-Analyses (PRISMA) guideline. Random-effects models were used to estimate the pooled percentage and its 95% CI. Three researchers performed the data extraction independently.

**Main Outcomes and Measures:**

The percentage of asymptomatic infections among the tested and confirmed populations.

**Results:**

Ninety-five unique eligible studies were included, covering 29 776 306 individuals undergoing testing. The pooled percentage of asymptomatic infections among the tested population was 0.25% (95% CI, 0.23%-0.27%), which was higher in nursing home residents or staff (4.52% [95% CI, 4.15%-4.89%]), air or cruise travelers (2.02% [95% CI, 1.66%-2.38%]), and pregnant women (2.34% [95% CI, 1.89%-2.78%]). The pooled percentage of asymptomatic infections among the confirmed population was 40.50% (95% CI, 33.50%-47.50%), which was higher in pregnant women (54.11% [95% CI, 39.16%-69.05%]), air or cruise travelers (52.91% [95% CI, 36.08%-69.73%]), and nursing home residents or staff (47.53% [95% CI, 36.36%-58.70%]).

**Conclusions and Relevance:**

In this meta-analysis of the percentage of asymptomatic SARS-CoV-2 infections among populations tested for and with confirmed COVID-19, the pooled percentage of asymptomatic infections was 0.25% among the tested population and 40.50% among the confirmed population. The high percentage of asymptomatic infections highlights the potential transmission risk of asymptomatic infections in communities.

## Key Points

**Question:**

What is the percentage of asymptomatic individuals with positive test results for SARS-CoV-2 among tested individuals and those with confirmed COVID-19 diagnosis?

**Findings:**

In this systematic review and meta-analysis of 95 unique studies with 29 776 306 individuals undergoing testing, the pooled percentage of asymptomatic infections was 0.25% among the tested population and 40.50% among the population with confirmed COVID-19.

**Meaning:**

The high percentage of asymptomatic infections from this study highlights the potential transmission risk of asymptomatic infections in communities.

## Teaser

This systematic review and meta-analysis evaluated the percentage of asymptomatic COVID-19 infections among individuals undergoing testing and those with confirmed infections.

## Introduction

COVID-19, the disease caused by SARS-CoV-2, was first reported in December 2019.^1^ Globally, as of January 28, 2021, there have been 100 455 529 confirmed cases, including 2 166 440 deaths.^2^ The disease course of COVID-19 ranges from asymptomatic to mild respiratory infections to pneumonia and even to acute respiratory distress syndrome.^3^ Patients with no symptoms at screening point were defined as having asymptomatic infections, which included infected people who have not yet developed symptoms but go on to develop symptoms later (presymptomatic infections), and those who are infected but never develop any symptoms (true asymptomatic or covert infections).^4,5^ Owing to the absence of symptoms, these patients would not seek medical care and could not be detected by temperature screening. Presymptomatic transmission will also make temperature screening less effective.^6^ Only extensive testing and close contact tracing could lead to identification of more asymptomatic infections.^7^

Unlike SARS, which had little known transmission from asymptomatic patients, evidence showed that asymptomatic patients were a potential source of transmission of COVID-19.^3,6^ A previous study^8^ showed that the upper respiratory viral loads in asymptomatic patients were comparable to those in symptomatic patients. Meanwhile, the highest viral load in throat swabs at the time of symptom onset indicated that infectiousness peaked on or before symptom onset.^9^ Moreover, studies showed that asymptomatic infections might have contributed to transmission among households, nursing facilities, and clusters.^10,11,12,13^ As the pandemic has been contained in many countries and regions, travel restrictions have been lifted and public places have reopened. Asymptomatic infections should be considered a source of COVID-19 infections that play an important role in the spread of the virus within community as public life gradually returns to normal. The management of asymptomatic carriers was essential for preventing cluster outbreaks and transmission within a community.

However, comprehensive evaluation of the percentage of asymptomatic infections among the tested population and the population with confirmed COVID-19 (confirmed population) is limited. Current results from different studies^3,5,7,8,10,11^ varied considerably owing to different study design and study population. Thus, we conducted a meta-analysis to better understand the global percentage of asymptomatic infections among the tested and confirmed COVID-19 populations. Our results could be useful for strategies to reduce transmission by asymptomatic infections.

## Methods

### Search Strategy

We conducted the meta-analysis following the Preferred Reporting Items for Systematic Reviews and Meta-Analyses (PRISMA) guideline. This review was not registered. Three researchers (Q.L., L.K., and R.L.) searched the published studies on February 4, 2021, through PubMed, EMBASE, and ScienceDirect without language restriction. The search terms used included *COVID-19*, *coronavirus*, *SARS-CoV-2*, *asymptomatic transmission*, *asymptomatic infection*, *asymptomatic proportion*, *asymptomatic case*, *asymptomatic cases*, *asymptomatic contact*, *asymptomatic ratio*, *asymptomatic people*, *asymptomatic patients*, and *asymptomatic patient*. The detailed search strategies are shown in eMethods 1 in the Supplement. Three researchers (Q.L., L.K., and R.L.) reviewed the titles, abstracts, and full texts of articles independently and identified additional studies from the reference lists. Disagreements were resolved by 2 other reviewers (W.J. and Y.W.).

### Selection Criteria

Asymptomatic individuals with positive test results for SARS-CoV-2 (asymptomatic infections) were defined as those who did not present any symptoms at the time of SARS-CoV-2 testing or diagnosis.^14^ Individuals with a confirmed COVID-19 diagnosis were defined as those who had a throat swab or other specimen with positive results for SARS-CoV-2 using a real-time reverse-transcription polymerase chain reaction assay. Inclusion criteria consisted of (1) studies reporting the number of asymptomatic infections, tested population, and confirmed population and (2) cross-sectional studies, cohort studies, case series studies, and case series on transmission. Exclusion criteria consisted of (1) reviews, systematic reviews, and meta-analysis; (2) duplicate publications; (3) preprints; (4) multiple studies reporting on overlapping participants (the study with more information was included); (5) articles with ambiguous definition of asymptomatic infections; and (6) articles not written in English or Chinese.

### Data Extraction and Quality Assessment

Three researchers (Q.L., L.K., and R.L.) performed the data extraction independently. Data were extracted for the first author, date of publication, study location, number of tested individuals, number of individuals with confirmed COVID-19, and number of asymptomatic infections. The ratio of male to female individuals (MFR) and mean age of study participants were gathered if available. The quality of studies included in the meta-analysis was assessed using the Joanna Briggs Institute Prevalence Critical Appraisal Tool^15^ for cross-sectional studies and the Newcastle-Ottawa scale^16^ for cohort studies (eMethods 2 in the Supplement). Case series on transmission were assessed using the quality assessment tool developed by Yanes-Lane et al.^17^ Two researchers (Q.L. and L.K.) performed the quality assessment independently. Disagreements were resolved by 2 other reviewers (W.J. and Y.W.). Outcomes of interest included the percentages of asymptomatic infections among the tested and the confirmed populations.

### Statistical Analysis

We performed a meta-analysis to estimate the pooled percentage of asymptomatic infections among the tested and confirmed populations. Untransformed percentages and DerSimonian and Laird random-effects models^18^ were used to calculate the pooled percentage and its 95% CI. The heterogeneity among studies was assessed using *I*^2^ values.^19^ We performed subgroup analyses by study location (Africa, Asia, Europe, North America, and South America), countries’ development level (developed vs developing), study population (air or cruise travelers, close contact, community residents, health care workers or in-hospital patients, nursing home residents or staff, and pregnant women), publication period (June 2020 and earlier vs July 2020 and later), sample size for the tested population (1-99, 100-999, 1000-9999, and ≥10 000), sample size for the confirmed population (1-99, 100-499, and ≥500), study design (case series, case series on transmission, cohort studies, and cross-sectional studies), study quality (low, moderate, and high), MFR (0 to <0.5, 0.5 to <1.0, 1.0 to <1.5, and ≥1.5), and mean age (<20, 20-39, 40-59, and ≥60 years). Publication bias was assessed by funnel plot and the Egger regression test.^20^ We performed 3 sensitivity analyses to test the robustness of our results, by using the Knapp-Hartung adjustments^21^ to calculate the 95% CIs around the pooled effects, by excluding 3 studies with a tested population more than 200 000 and studies with low quality. Two-sided *P* < .05 indicated statistical significance. All analyses were performed using R, version 4.0.0 (R Project for Statistical Computing).

## Results

**Figure 1.**
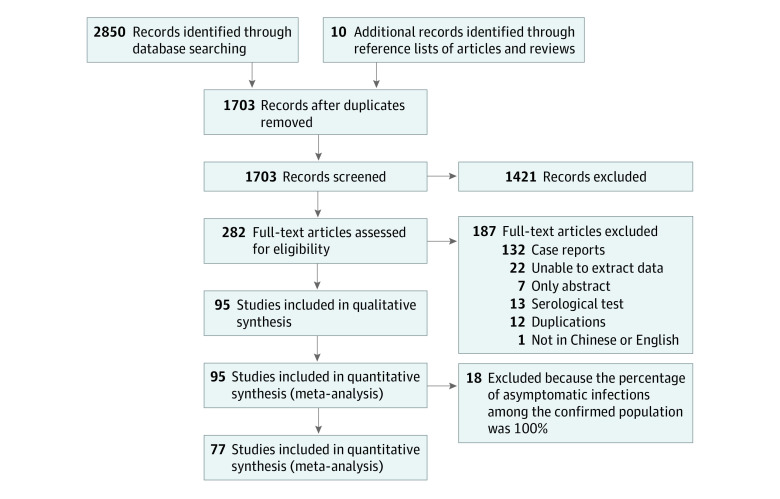
Flow Diagram of Study Selection

We identified 2860 studies through database search and the reference lists of articles and reviews. Of these, 282 studies underwent full-text review. Ninety-five studies with information concerning the percentage of asymptomatic infections among the tested and confirmed populations were included in the final analysis^12,22,23,24,25,26,27,28,29,30,31,32,33,34,35,36,37,38,39,40,41,42,43,44,45,46,47,48,49,50,51,52,53,54,55,56,57,58,59,60,61,62,63,64,65,66,67,68,69,70,71,72,73,74,75,76,77,78,79,80,81,82,83,84,85,86,87,88,89,90,91,92,93,94,95,96,97,98,99,100,101,102,103,104,105,106,107,108,109,110,111,112,113,114,115^ (Figure 1).

Among these studies, 44 (46.32%) were cross-sectional studies, 41 (43.16%) were cohort studies, 7 (7.37%) were case series, and 3 (3.16%) were case series on transmission studies. Thirty-five studies (36.84%) were conducted in Europe; 32 (33.68%), in North America; and 25 (26.32%), in Asia. Seventy-four studies (77.89%) were conducted in developed countries. Thirty-seven studies (38.95%) were conducted among health care workers or in-hospital patients; 17 (17.89%), among nursing home residents or staff; 14 (14.74%), among community residents; 13 (13.68%), among pregnant women; 8 (8.42%), among air or cruise travelers; and 6 (6.32%), among close contacts. Twenty-one studies (22.11%) were published in June or before; 74 (77.89%), in July and after. Forty-nine studies (51.58%) had sample size of 100 to 1000. Fifty-three studies (55.79%) were assessed as low quality; 17 (17.89%), high quality; and 25 (26.32%), moderate quality (Table). For cross-sectional studies, low-quality studies were mostly those without random sampling or with 2 or more biases (selection bias, reporting bias, or detection bias). For cohort studies, low-quality studies were mostly those with 1 or more biases.

**Table.** Characteristics of the Studies Included for Meta-analysis

Source	Country	Study design	Time of publication	Population group	No. tested individuals	No. confirmed individuals	No. asymptomatic infections	Quality
Abdelmoniem et al^22^	Egypt	Cross-sectional	January 2020	Health care workers or in-hospital patients	203	29	29	Low
Abeysuriya et al^23^	UK	Cross-sectional	September 2020	Pregnant women	180	7	6	Low
Akbarialiabad et al^24^	Iran	Cross-sectional	September 2020	Health care workers or in-hospital patients	1805	86	19	Low
Al-Qahtani et al^25^	Kingdom of Bahrain	Cohort	November 2020	Air or cruise travelers	2714	188	116	High
Al-Shamsi et al^26^	United Arab Emirates	Cohort	November 2020	Health care workers or in-hospital patients	109	32	6	Low
Arnold et al^27^	US	Cross-sectional	January 2021	Health care workers or/in-hospital patients	2882	103	38	Moderate
Arons et al^12^	US	Cross-sectional	April 2020	Nursing home residents or staff	76	48	27	Moderate
Aslam et al^28^	US	Cohort	January 2020	Health care workers or in-hospital patients	11 622	69	42	Low
Bayle et al^29^	France	Cross-sectional	January 2021	Nursing home residents or staff	241	32	24	Moderate
Bender et al^30^	US	Cohort	September 2020	Pregnant women	318	8	8	Moderate
Bianco et al^31^	US	Cross-sectional	May 2020	Pregnant women	155	24	24	Low
Blain et al^32^	US	Case series	July 2020	Nursing home residents or staff	113	44	8	Moderate
Blitz et al^33^	US	Cohort	August 2020	Pregnant women	382	71	45	Low
Blumberg et al^34^	US	Cohort	October 2020	Health care workers or in-hospital patients	1198	7	6	Low
Bosworth et al^35^	UK	Cross-sectional	July 2020	Health care workers or in-hospital patients	1282	53	16	Moderate
Cao et al^36^	China	Cross-sectional	November 2020	Community residents	9 865 404	300	300	High
Carroll et al^37^	Ireland	Cohort	October 2020	Close contact	4586	310	209	Moderate
Cattelan et al^38^	Italy	Cohort	August 2020	Health care workers or in-hospital patients	7595	395	109	Low
Cloutier et al^39^	Canada	Cross-sectional	August 2020	Community residents	330	6	6	Low
Corcorran et al^40^	US	Cohort	August 2020	Health care workers or in-hospital patients	25	10	4	Low
Deng et al^41^	China	Case series on transmission	October 2020	Close contact	347	27	1	High
Dora et al^42^	US	Cross-sectional	May 2020	Nursing home residents or staff	235	27	18	Low
Duan et al^43^	China	Cross-sectional	September 2020	Health care workers or/in-hospital patients	4729	4	4	Moderate
Figueiredo et al^44^	Portugal	Cohort	October 2020	Pregnant women	184	11	9	Low
Goldfarb et al^45^	US	Cross-sectional	May 2020	Pregnant women	757	20	9	Moderate
Graham et al^46^	UK	Cross-sectional	September 2020	Nursing home residents or staff	464	129	54	Moderate
Grechukhina et al^47^	US	Cohort	November 2020	Pregnant women	1567	141	44	High
Gruskay et al^48^	US	Cohort	June 2020	Health care workers or in-hospital patients	99	12	7	Low
Han et al^49^	China	Cross-sectional	June 2020	Community residents	29 299	18	18	Low
Harada et al^50^	Japan	Cohort	December 2020	Health care workers or in-hospital patients	1259	79	33	Low
Hcini et al^51^	France	Cohort	February 2020	Pregnant women	507	137	103	Low
Hoxha et al^52^	Belgium	Cross-sectional	July 2020	Nursing home residents or staff	280 427	8325	6244	Moderate
Hung et al^53^	China	Case series	September 2020	Air or cruise travelers	215	9	6	High
Ibrahim et al^54^	Indonesia	Case series	August 2020	Health care workers or in-hospital patients	4617	582	55	Low
Kennelly et al^55^	Ireland	Cohort	September 2020	Nursing home residents or staff	2968	1105	290	Low
Kessler et al^56^	Germany	Cross-sectional	December 2020	Health care workers or in-hospital patients	689	1	1	Moderate
Kimball et al^57^	US	Cross-sectional	April 2020	Nursing home residents or staff	76	23	13	Moderate
Kirshblum et al^58^	US	Cohort	July 2020	Health care workers or in-hospital patients	103	12	12	Low
Krüger et al^59^	Germany	Cohort	January 2021	Health care workers or in-hospital patients	6940	27	7	Low
Kwon et al^60^	South Korea	Cross-sectional	July 2020	Health care workers or in-hospital patients	2087	42	6	Low
LaCourse et al^61^	US	Cohort	May 2020	Pregnant women	230	13	1	Low
Ladhani et al^62^	UK	Cohort	September 2020	Nursing home residents or staff	518	158	97	High
Lan et al^63^	US	Cross-sectional	November 2020	Community residents	104	21	16	Moderate
Lavezzo et al^64^	Italy	Cross-sectional	July 2020	Community residents	2812	73	29	Moderate
Livingston et al^65^	UK	Cohort	October 2020	Health care workers or in-hospital patients	344	131	16	Moderate
Lombardi et al^66^	Italy	Cohort	June 2020	Health care workers or in-hospital patients	1573	139	28	Low
Ly et al^67^	France	Cross-sectional	November 2020	Nursing home residents or staff	1691	226	46	Moderate
Lytras et al^68^	Greece	Cross-sectional	April 2020	Air or cruise travelers	783	40	35	Low
Maechler et al^69^	Germany	Cross-sectional	December 2020	Community residents	4333	333	14	High
Marossy et al^70^	UK	Cross-sectional	September 2020	Nursing home residents or staff	2455	160	115	Moderate
Marschner et al^71^	Germany	Cross-sectional	July 2020	Health care workers or in-hospital patients	139	1	1	Low
Martinez-Fierro et al^72^	Mexico	Cross-sectional	October 2020	Close contact	81	34	5	Low
Massarotti et al^73^	Italy	Cross-sectional	August 2020	Pregnant women	333	7	6	Low
Mattar et al^74^	Caribbean	Cross-sectional	December 2020	Close contact	686	35	18	Low
Menting et al^75^	Germany	Cross-sectional	January 2020	Health care workers or in-hospital patients	1185	11	2	Low
Migueres et al^76^	France	Cross-sectional	September 2020	Health care workers or in-hospital patients	123	44	17	Low
Milani et al^77^	Italy	Cross-sectional	June 2020	Community residents	197	21	21	Moderate
Nishiura et al^78^	Japan	Cross-sectional	May 2020	Air or cruise travelers	565	13	4	Low
Ochiai et al^79^	Japan	Cross-sectional	June 2020	Pregnant women	52	2	2	Low
Olalla et al^80^	Spain	Cross-sectional	August 2020	Health care workers or in-hospital patients	498	2	2	Low
Olmos et al^81^	Chile	Cross-sectional	January 2021	Health care workers or in-hospital patients	413	14	14	Low
Park et al^82^	South Korea	Cross-sectional	April 2020	Community residents	1143	97	8	High
Park et al^83^	Korea	Cohort	December 2020	Air or cruise travelers	39	30	4	Low
Patel et al^84^	United States	Cohort	June 2020	Nursing home residents or staff	126	35	14	Low
Pavli et al^85^	Greece	Case series on transmission	September 2020	Air or cruise travelers	891	5	2	High
Petersen et al^86^	United Kingdom	Cross-sectional	October 2020	Community residents	36 061	115	88	Moderate
Puckett et al^87^	United States	Cohort	December 2020	Health care workers or in-hospital patients	227	2	2	Low
Ralli et al^88^	Italy	Cohort	December 2020	Community residents	298	12	9	Low
Rashid-Abdi et al^89^	Sweden	Cohort	November 2020	Health care workers or in-hospital patients	131	21	1	Low
Ren et al^90^	China	Cohort	February 2021	Air or cruise travelers	19 398 384	3103	1749	High
Rincón et al^91^	Spain	Cohort	September 2020	Health care workers or in-hospital patients	192	36	14	Low
Roxby et al^92^	United States	Cohort	May 2020	Nursing home residents or staff	80	3	2	Low
Sacco et al^93^	France	Cohort	November 2020	Nursing home residents or staff	179	63	12	Low
Santos et al^94^	Portugal	Cross-sectional	December 2020	Health care workers or in-hospital patients	8037	211	47	Low
Scheier et al^95^	Switzerland	Cross-sectional	February 2021	Health care workers or in-hospital patients	2807	68	8	High
Shah et al^96^	US	Case series	July 2020	Health care workers or in-hospital patients	625	1	1	Low
Shi et al^97^	US	Cohort	October 2020	Nursing home residents or staff	389	146	66	Moderate
Singer et al^98^	US	Case series	October 2020	Health care workers or in-hospital patients	4751	18	10	High
Tang et al^99^	China	Cross-sectional	July 2020	Health care workers or in-hospital patients	1027	52	13	High
Tang et al^100^	US	Cohort	November 2020	Nursing home residents or staff	1970	752	424	High
Temkin et al^101^	Israel	Cross-sectional	October 2020	Health care workers or in-hospital patients	522	1	1	Low
Trahan et al^102^	Canada	Cohort	November 2020	Pregnant women	803	41	11	Low
Tsou et al^103^	China	Case series	November 2020	Community residents	17 935	100	10	Moderate
van Buul et al^104^	The Netherlands	Cohort	Decem ber 2020	Nursing home residents or staff	839	25	6	High
Varnell et al^105^	US	Cohort	January 2021	Health care workers or in-hospital patients	281	24	9	Moderate
Wadhwa et al^106^	US	Cohort	December 2020	Community residents	172	19	12	Moderate
Wi et al^107^	South Korea	Case series	July 2020	Community residents	17 400	111	25	High
Wood et al^108^	Indiana	Cross-sectional	August 2020	Community residents	511	1	1	Low
Yamahata et al^109^	Japan	Cross-sectional	May 2020	Air or cruise travelers	3711	696	410	Moderate
Yassa et al^110^	Turkey	Cohort	July 2020	Pregnant women	296	23	12	Low
Yau et al^111^	Canada	Cohort	July 2020	Health care workers or in-hospital patients	330	22	12	Low
Yousaf et al^112^	US	Cohort	July 2020	Close contact	195	47	6	Low
Zhang et al^113^	China	Case series on transmission	April 2020	Close contact	8437	25	3	High
Zhang et al^114^	China	Cohort	September 2020	Health care workers or in-hospital patients	8553	235	21	Low
Zhao et al^115^	China	Cohort	August 2020	Health care workers or in-hospital patients	1060	160	38	Low

### Percentage of Asymptomatic Infections Among the Tested Population

Ninety-five studies were included in the meta-analysis for the percentage of asymptomatic infections among the tested population, covering 29 776 306 tested individuals, among whom 11 516 had asymptomatic infections. The pooled percentage of asymptomatic infections among the tested population was 0.25% (95% CI, 0.23%-0.27%), with high heterogeneity among studies (*I*^2^ = 99%; *P* < .001) (eFigure 1 in the Supplement).

**Figure 2.**
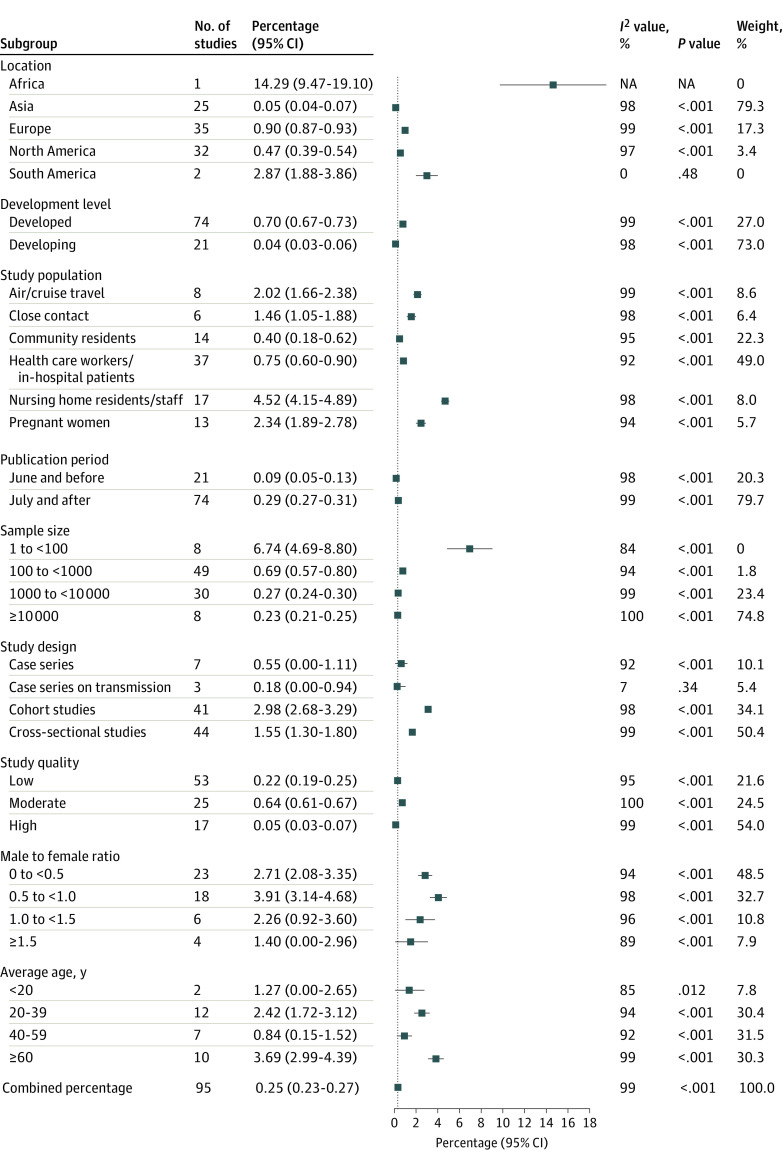
Percentage of Asymptomatic Infections Among the Tested Population by Subgroups Includes 29 776 306 tested individuals, among whom 11 516 had asymptomatic infections.

Among tested individuals in different study populations, the pooled percentage of asymptomatic infections was 4.52% (95% CI, 4.15%-4.89%) in nursing home residents or staff, 2.02% (95% CI, 1.66%-2.38%) in air or cruise travelers, 2.34% (95% CI, 1.89%-2.78%) in pregnant women, 1.46% (95% CI, 1.05%-1.88%) in close contacts, 0.75% (95% CI, 0.60%-0.90%) in health care workers or in-hospital patients, and 0.40% (95% CI, 0.18%-0.62%) in community residents. The pooled percentage of asymptomatic infections was 0.90% (95% CI, 0.87%-0.93%) in Europe, 0.47% (95% CI, 0.39%-0.54%) in North America, and 0.05% (95% CI, 0.04%-0.07%) in Asia. The pooled percentage was higher in developed countries (0.70% [95% CI, 0.67%-0.73%]), studies published in July or later (0.29% [95% CI, 0.27%-0.31%]), studies with a sample size of less than 100 (6.74% [95% CI, 4.69%-8.80%]), and cohort studies (2.98% [95% CI, 2.68%-3.29%]). In studies with MFR of 0.5 to less than 1.0, the pooled percentage was higher (3.91%; [95% CI, 3.14%-4.68%]). The pooled percentage was higher when the mean age of the study population was 60 years or older (3.69% [95% CI, 2.99%-4.39%]) (Figure 2).

### Percentage of Asymptomatic Infections Among the Confirmed Population

Among 95 studies, 18 were excluded because that the percentage of asymptomatic infections among the confirmed population was 100%.^22,30,31,36,39,43,49,56,58,71,77,79,80,81,87,96,101,108^ The remaining 77 studies were included in the meta-analysis for the percentage of asymptomatic infections among the confirmed population,^12,23,24,25,26,27,28,29,32,33,34,35,37,38,40,41,42,44,45,46,47,48,50,51,52,53,54,55,57,59,60,61,62,63,64,65,66,67,68,69,70,72,73,74,75,76,78,82,83,84,85,86,88,89,90,91,92,93,94,95,97,98,99,100,102,103,104,105,106,107,109,110,111,112,113,114,115^ covering 19 884 individuals with confirmed COVID-19, among whom 11 069 had asymptomatic infections. The pooled percentage of asymptomatic infections among the confirmed population was 40.50% (95% CI, 33.50%-47.50%), with high heterogeneity among studies (*I*^2^ = 99%; *P* < .001) (eFigure 2 in the Supplement).

**Figure 3.**
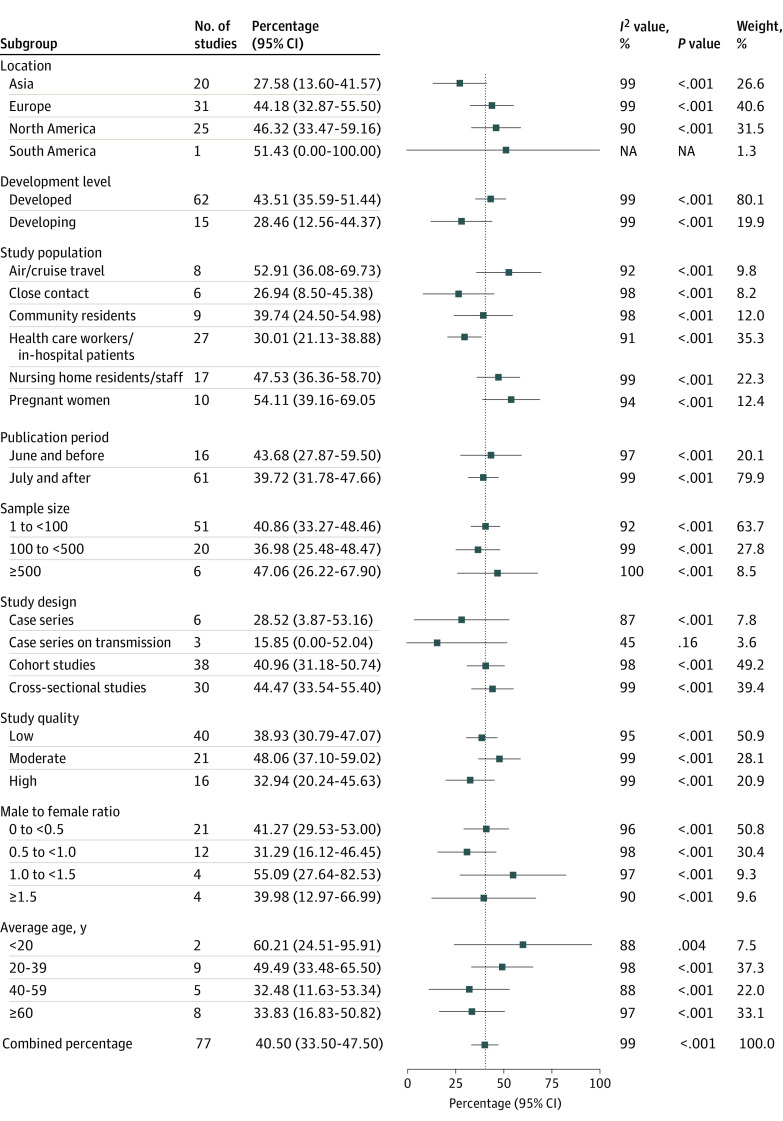
Percentage of Asymptomatic Infections Among the Confirmed Population by Subgroups Includes 19 884 individuals with confirmed COVID-19, among whom 11 069 had asymptomatic infections.

Among the confirmed population, the pooled percentage of asymptomatic infections was 54.11% (95% CI, 39.16%-69.05%) in pregnant women, 52.91% (95% CI, 36.08%-69.73%) in air or cruise travelers, 47.53% (95% CI, 36.36%-58.70%) in nursing home residents or staff, 39.74% (95% CI, 24.50%-54.98%) in community residents, 30.01% (95% CI, 21.13%-38.88%) in health care workers or in-hospital patients, and 26.94% (95% CI, 8.50%-45.38%) in close contacts. The pooled percentage of asymptomatic infections was 46.32% (95% CI, 33.47%-59.16%) in North America, 44.18% (95% CI, 32.87%-55.50%) in Europe, and 27.58% (95% CI, 13.60%-41.57%) in Asia. The pooled percentage was higher in developed countries (43.51% [95% CI, 35.59%-51.44%]), studies published in June or earlier (43.68% [95% CI, 27.87%-59.50%]), studies with sample size of 500 or greater (47.06% [95% CI, 26.22%-67.90%]), and cross-sectional studies (44.47% [95% CI, 33.54%-55.40%]). The pooled percentage was slightly lower for cohort studies (40.96% [95% CI, 31.18%-50.74%]). Among studies with MFR of 1.0 to less than 1.5, the pooled percentage was higher (55.09% [95% CI, 27.64%-82.53%]). The pooled percentage was higher when the mean age of the study population was younger than 20 years (60.21% [95% CI, 24.51%-95.91%]) or 20 to 39 years (49.49% [95% CI, 33.48%-65.50%]) (Figure 3).

### Sensitivity Analysis and Publication Bias

After using the Knapp-Hartung adjustments, the pooled percentage of asymptomatic infections among the tested population was 0.25% (95% CI, 0.11%-0.39%), and the 95% CI of the pooled percentage became slightly larger (eFigure 3 in the Supplement). The percentage of asymptomatic infections among the confirmed population was 40.50% (95% CI, 34.94%-46.07%), and the 95% CI of the pooled percentage became slightly narrower (eFigure 4 in the Supplement).

After excluding 3 studies with tested populations of more than 200 000,^36,52,90^ the pooled percentage of asymptomatic infections among the tested population was 1.61% (95% CI, 1.47%-1.76%), which was higher than the original results. The percentage of asymptomatic infections among the confirmed population was 39.37% (95% CI, 33.86%-44.87%), which was slightly lower than the original results. After excluding 53 low-quality studies, the pooled percentage of asymptomatic infections among the tested population was 0.24% (95% CI, 0.23%-0.26%), and the percentage of asymptomatic infections among the confirmed population was 41.71% (95% CI, 31.89%-51.53%). Both percentages were similar to the original results.

**Figure 4.**
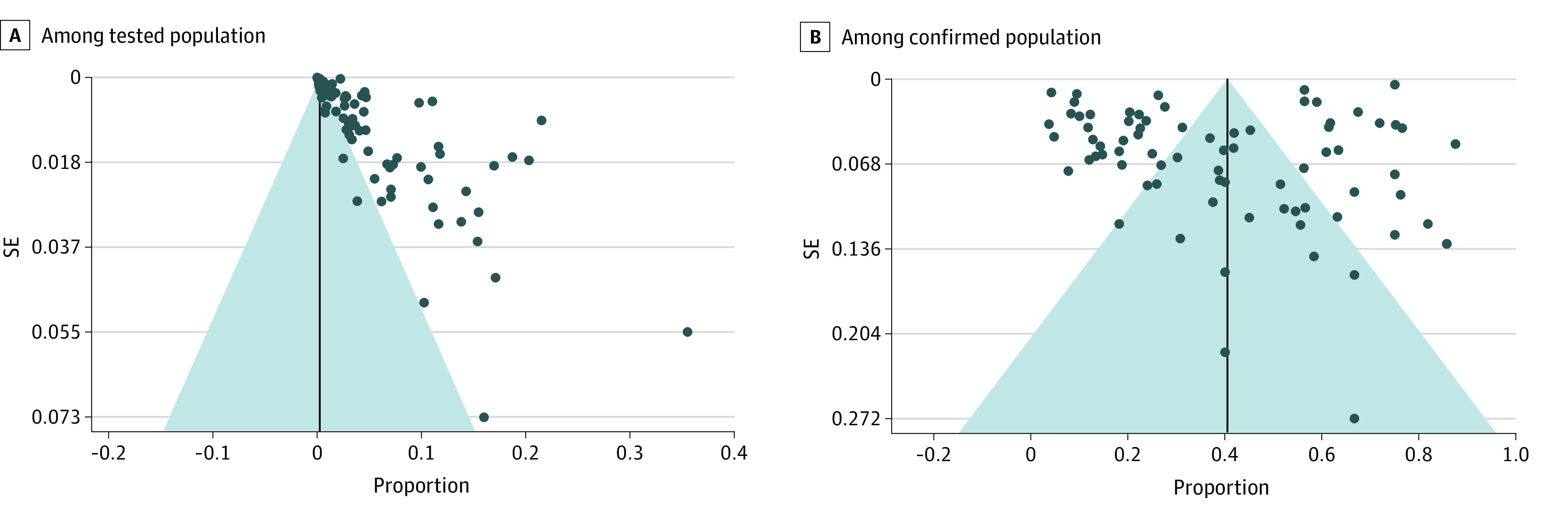
Funnel Plots Based on the Percentage of Asymptomatic Infections Includes 29 776 306 tested individuals, among whom 11 516 had asymptomatic infections and 19 884 individuals with confirmed COVID-19, among whom 11 069 had asymptomatic infections. Funnel plot asymmetry indicated possible publication bias.

Funnel plots are shown in Figure 4. Egger regression tests for the percentage of asymptomatic infections among the tested population (*z* = 43.1725; *P* < .001) and for the percentage of asymptomatic infections among the confirmed population (*z* = 2.3846; *P* = .02) indicated that there might be publication bias.

## Discussion

In this meta-analysis, we found that the pooled percentage of asymptomatic infections among the tested population was 0.25% (95% CI, 0.23%-0.27%), and the pooled percentage of asymptomatic infections among the confirmed population was 40.50% (95% CI, 33.50%-47.50%). At present, there are only a few meta-analyses for the percentage of asymptomatic infections among the tested population. We found that the percentage of asymptomatic infections was highest among the tested population in nursing homes and lowest among community residents. Because the percentage of asymptomatic individuals varies as a function of community prevalence, it was not available in all studies. This might be a potential driver of heterogeneity across studies. Furthermore, the percentages of asymptomatic infections among the tested population were different between studies conducted in different locations. Studies in Asia had the lowest percentage, whereas studies in other locations had higher percentages. This lower percentage in Asia might be related to the large city-wide SARS-CoV-2 nucleic acid screening program in China.^36^ In the sensitivity analyses, we found that the pooled percentage of asymptomatic infections among the tested population was higher than the original results after excluding studies with large sample sizes. This indicated that studies with different sample sizes were very heterogeneous. Owing to severe outcomes among older patients with COVID-19, more studies were conducted among nursing home residents or staff. Thus, asymptomatic individuals were more likely to be tested among this population. As more and more countries conducted expanded screening, studies concerning the percentage of asymptomatic infections among the general population would increase in the future.

In this study, the pooled percentage of asymptomatic infections among the confirmed population was 40.50%. The pooled percentage of asymptomatic infections was 40.96% among cohort studies, which was slightly lower than that among cross-sectional studies (44.47%). The patients who developed symptoms later were mistakenly classified as having asymptomatic infection in cross-sectional studies because the observation time was not long enough.^14^ Thus, the percentage of asymptomatic infections was lower in cohort studies, because some patients with presymptomatic findings were identified during follow-up. There were limited case series of great interest in the first months of the pandemic; however, these studies mostly traced and tested limited contacts, which contributed limited value to the evidence of the percentage of asymptomatic infections.^17^ Several meta-analyses concerned the percentage of asymptomatic infections among the confirmed population. Chen et al^5^ conducted a meta-analysis that included 104 published studies and preprints before May 13, 2020. They found that the percentage of asymptomatic individuals among those with COVID-19 was 13.34% (95% CI, 10.86%-16.29%). Unlike our study, Chen et al^5^ searched a Chinese database. Thus, the percentage of Chinese studies was higher in their study than in the present study. He et al^14^ searched PubMed and Embase before May 20, 2020, and included 41 published studies. More than 50% of the studies were from China, and the pooled percentage of asymptomatic infection was 15.6% (95% CI, 10.1%-23.0%). In our study, we only included published studies. The percentage of countries excluding China was higher than the previous meta-analysis.^14^ This might be the reason for the higher percentage of asymptomatic infections found in our study compared with studies conducted by Chen et al^5^ and He et al.^14^ Another meta-analysis conducted by Yanes-Lane et al^17^ included published studies and preprints before June 22, 2020. After quality assessment, 28 studies were of high or moderate quality and were included in the meta-analysis. The percentage of asymptomatic infection among persons with confirmed COVID-19 varied among different study populations, with the highest observed in obstetric patients (95% [95% CI, 45%-100%]).

In our study, the percentage of asymptomatic infections among the confirmed population was 54.11% in pregnant women and 52.91% in air or cruise travelers. The percentage was 47.53% in nursing home residents or staff. This finding of a high percentage of asymptomatic infections among air or cruise travelers suggests that screening and quarantine on airport arrival is important for reducing community transmissions, especially in countries without local transmission.^3,25^ In addition, we found that the percentage of asymptomatic infections among the tested population was relatively low among community residents. However, the percentage of asymptomatic infection among confirmed individuals was 39.74% in communities. These findings suggest that asymptomatic infections might contribute to the transmission of SARS-CoV-2 within the community. To prevent further transmission in communities, asymptomatic individuals among the general population should be tested. If resources are limited, workers in specific industries such as air transportation should be routinely tested. In addition, we found that approximately one-third of individuals with confirmed COVID-19 were asymptomatic among health care workers or in-hospital patients. Because asymptomatic health care workers might contribute to disease spread in and out of hospitals, surveillance of asymptomatic individuals is important for infection control and transmission reduction in health care settings and community.^116,117^ Meanwhile, hand hygiene and personal protective equipment were necessary for hospital visitors.^117^ A previous study showed that most asymptomatic patients belong to younger groups,^3^ which was consistent with the findings of our study. The percentage of asymptomatic infections was higher among groups younger than 39 years than in other age groups, possibly because the young adults were more likely to show only mild or moderate clinical symptoms.^5^ This indicated that young adults who often presented mild or no symptoms were a potential source of transmission in the community.

In the meta-analysis, we included studies published before February 3, 2021, providing the most updated pooled percentage of asymptomatic infections among tested and confirmed populations. We included countries in Africa, Asia, Europe, North America, and South America and estimated the percentage of asymptomatic infections for different populations. Our results could raise awareness among the public and policy makers and provide evidence for prevention strategies.

### Limitations

This study has several limitations. First, we did not include preprints and therefore may have missed some relevant studies; however, we thought that the results of published studies were more reliable. Second, some relevant articles written in Chinese may not be included because we did not search Chinese literature databases such as China National Knowledge Infrastructure. Third, most studies did not follow up to identify presymptomatic and covert infections. Future studies should evaluate the percentage of these 2 types of asymptomatic infection among the confirmed population. Fourth, most studies were conducted in a specific population; thus, our findings might not be generalizable to the general population. Fifth, the heterogeneity between studies was high, which might be related to different study location, period, population, and sample size. Sixth, the Egger regression test suggested potential publication bias in this study. Because studies that did not detect asymptomatic infections were less likely to be published, our pooled percentage of asymptomatic infections might be overestimated.

## Conclusions

In this systematic review and meta-analysis, we found that the pooled percentage of asymptomatic SARS-CoV-2 infections among the tested population was 0.25%. Among the confirmed population, 40.50% of individuals had asymptomatic infections. The high percentage of asymptomatic infections highlights the potential transmission risk of asymptomatic infections in communities. Screening for asymptomatic infection is required, especially for countries and regions that have successfully controlled SARS-CoV-2. Asymptomatic infections should be under management similar to that for confirmed infections, including isolating and contact tracing.

## Notes

Supplement. **eMethods 1.** Search Strategies **eMethods 2.** Study Quality Assessments **eFigure 1.** Percentage of Asymptomatic Cases Among the Tested Population **eFigure 2.** Percentage of Asymptomatic Cases Among the Confirmed Population **eFigure 3.** Percentage of Asymptomatic Cases Among the Tested Population by Subgroups, Using the Knapp-Hartung Adjustments **eFigure 4.** Percentage of Asymptomatic Cases Among the Confirmed Population by Subgroups, Using the Knapp-Hartung Adjustments
