# Supplementary material for: Global Percentage of Asymptomatic SARS-CoV-2 Infections Among the Tested Population and Individuals With Confirmed COVID-19 Diagnosis: A Systematic Review and Meta-analysis
Source: JAMA Netw Open. 2021 Dec 14;4(12):e2137257. doi: 10.1001/jamanetworkopen.2021.37257 (PMC8672238; doi:10.1001/jamanetworkopen.2021.37257)
Supplement: Supplement. — eMethods 1. Search Strategies eMethods 2. Study Quality Assessments eFigure 1. Percentage of Asymptomatic Cases Among the Tested Population eFigure 2. Percentage of Asymptomatic Cases Among the Confirmed Population eFigure 3. Percentage of Asymptomatic Cases Among the Tested Population by Subgroups, Using the Knapp-Hartung Adjustments eFigure 4. Percentage of Asymptomatic Cases Among the Confirmed Population by Subgroups, Using the Knapp-Hartung Adjustments [file jamanetwopen-e2137257-s001.pdf]

Supplementary Online Content

Ma Q, Liu J, Liu Q, et al. Global percentage of asymptomatic SARS-CoV-2 infections among the tested population and individuals with confirmed COVID-19 diagnosis: a systematic review and meta-analysis. *JAMA Netw Open*. 2021;4(12):e2137257. doi:10.1001/jamanetworkopen.2021.37257

eMethods 1. Search Strategies
eMethods 2. Study Quality Assessments
eFigure 1. Percentage of Asymptomatic Cases Among the Tested Population
eFigure 2. Percentage of Asymptomatic Cases Among the Confirmed Population
eFigure 3. Percentage of Asymptomatic Cases Among the Tested Population by Subgroups, Using the Knapp-Hartung Adjustments
eFigure 4. Percentage of Asymptomatic Cases Among the Confirmed Population by Subgroups, Using the Knapp-Hartung Adjustments

This supplementary material has been provided by the authors to give readers additional information about their work.

eMethods 1. Search Strategies

PubMed

("COVID-19"[Title/Abstract] OR "coronavirus"[Title/Abstract] OR "SARS-CoV-2"[Title/Abstract]) AND ("asymptomatic transmission"[Title/Abstract] OR "asymptomatic infection"[Title/Abstract] OR "asymptomatic proportion"[Title/Abstract] OR "asymptomatic carrier"[Title/Abstract] OR "asymptomatic carriers"[Title/Abstract] OR "asymptomatic contact"[Title/Abstract] OR "asymptomatic ratio"[Title/Abstract] OR "asymptomatic cases"[Title/Abstract] OR "asymptomatic case"[Title/Abstract] OR "asymptomatic people"[Title/Abstract] OR "asymptomatic patients"[Title/Abstract] OR "asymptomatic patient"[Title/Abstract]) AND 2019/11/01:2021/12/27[Date - Publication]

EMBASE

('covid 19':ab,ti OR 'sars cov 2':ab,ti OR coronavirus:ab,ti) AND ('asymptomatic transmission':ab,ti OR 'asymptomatic infection':ab,ti OR 'asymptomatic proportion':ab,ti OR 'asymptomatic carrier':ab,ti OR 'asymptomatic carriers':ab,ti OR 'asymptomatic contact':ab,ti OR 'asymptomatic ratio':ab,ti OR 'asymptomatic cases':ab,ti OR 'asymptomatic case':ab,ti OR 'asymptomatic people':ab,ti OR 'asymptomatic patients':ab,ti OR 'asymptomatic patient':ab,ti) AND [1-11-2019]/sd NOT [28-12-2020]/sd

ScienceDirect

((COVID-19)OR(coronavirus)OR(SARS-CoV-2))+ ((asymptomatic transmission)OR(asymptomatic infection)OR(asymptomatic proportion)OR(asymptomatic carrier)OR(asymptomatic contact)OR(asymptomatic ratio)OR(asymptomatic case)OR(asymptomatic people)OR(asymptomatic patient))

((COVID-19)OR(coronavirus)OR(SARS-CoV-2))+ ((asymptomatic carriers)OR(asymptomatic cases)OR(asymptomatic patients))

© 2021 Ma Q et al. *JAMA Network Open*.

eMethods 2. Study Quality Assessments (according to the quality assessment by Yanes-Lane et al[18])

Cohort studies

1.1 Is the cohort representative of the target population?	1.2 Is there likely selection bias?	2.1 Was there a clear definition for an asymptomatic case?	2.2 Is there likely reporting bias?	3.1 Were objective, standard criteria used for diagnosis of the condition?	3.2 Was symptom development assessed in asymptomatic subjects?	3.3 Was follow-up long enough for symptoms to occur? (14 days on average)	3.4 Were symptoms assessed in a systematic and reliable way?	3.5 Adequacy of follow up of cohorts (lost to follow up)	3.6 Is there likely detection bias?

Grading scale: If question 1.2 is answered “yes” then the study is automatically low quality. If one question assessing bias (2.2 or 3.6) is answered "yes" then study is moderate quality. If both questions assessing bias (2.2 and 3.6) are answered "yes" then the study is low quality.

Cross-sectional studies

1.1 Were the criteria for inclusion in the sample clearly defined?	1.2 Was an appropriate method of sampling used? Random, complete, other.	1.3 Participation rate	1.4 Were the study subjects representative of the target population?	1.5 Is there likely to be selection bias?	2.1 Was there a clear definition for an asymptomatic case?	2.2 Were the study subjects and the setting described in detail?	2.3 Is there likely to be reporting bias?	3.1 Were objective, standard criteria used for diagnosis of the condition?	3.2 Was symptom assessment carried out in a standard objective way?	3.3 Is there likely detection bias?

Grading scale: If questions 1.2 is not a random sample, or if question 1.3 has a lower than 70% participation rate, then the study is automatically low quality. If one question assessing bias (1.5, 2.3 or 3.3) is answered "yes" then study is moderate quality. If two or more questions assessing bias (1.5, 2.3 or 3.3) are answered "yes" then the study is low quality.

© 2021 Ma Q et al. *JAMA Network Open*.

Case series

1.1 Were the inclusion criteria for the case series clearly defined?	1.2 Were valid methods used to diagnose COVID-19 for all participants included in the case series?	1.3 Did the case series have consecutive inclusion of participants?	1.4 Did the case series have complete inclusion of participants?	1.5 Is there likely selection bias ?	2.1 Was there clear reporting of the demographics of the participants in the study?	2.2 Was there clear reporting of clinical information of the participants?	2.3 Were the outcomes or follow up results of cases clearly reported?	2.4 Was there clear reporting of the presenting site(s)/clinic(s) demographic information?	2.5 Is there likely reporting bias?

Grading scale: If question 1.1 is answered "no" then the study is automatically low quality, and if 1.1 is answered "yes" but questions 1.3 or 1.4 are answered "no" then the study is also low quality. If one question assessing bias (1.5 or 2.5) is answered "yes" then study is moderate quality. If both questions assessing bias (1.5 or 2.5) are answered "yes" then the study is low quality.

Case series on transmission

1.1 Is the method for contact tracing clearly defined?	1.2 Is the number of contacts identified and tested clearly reported?	1.3 Is there likely bias due to missing information? i.e reporting bias	2.1 Is the method for contact tracing rigorous and exhaustive to identify all potential contacts?	2.2 Was the follow-up of people without symptoms sufficient to allow for the incubation period?	2.3 Is there likely missing data on contacts ?	2.4 Is there likely bias in contact identification?	2.5 Is there likely exposure to other potential index cases that were symptomatic?	2.6 Was the index case symptomatic at any time during the exposure to contacts?	2.7 Was the index case identified after the contacts? i.e was the index case identified by exclusion	2.8 Is there likely bias in index case identificati on?

Grading scale: If questions 2.6 AND 2.7 are answered "yes" then the study is automatically low quality. If one question assessing bias (1.3, 2.4 or 2.8) is answered "yes" then study is moderate quality. If two or more signalling questions are answered “yes” then the study is low quality. For studies on household transmission, signalling questions 1.1 and 1.2 were omitted.

© 2021 Ma Q et al. *JAMA Network Open*.

eFigure 1. Percentage of Asymptomatic Cases Among the Tested Population

Study	Asymptomatic	Tested individuals	Percentage	95% CI	Weight
Cao et al (2020.11)	300	9865404	0.00	[0.00; 0.00]	17.1%
Ren et al (2021.2)	1749	19398384	0.01	[0.01; 0.01]	17.1%
Zhang et al (2020.4)	3	8437	0.04	[0.00; 0.08]	8.3%
Tsou et al (2020.11)	10	17935	0.06	[0.02; 0.09]	9.6%
Han et al (2020.6)	18	29299	0.06	[0.03; 0.09]	11.2%
Duan et al (2020.9)	4	4729	0.08	[0.00; 0.17]	3.1%
Krüger et al (2021.1)	7	6940	0.10	[0.03; 0.18]	3.7%
Wi et al (2020.7)	25	17400	0.14	[0.09; 0.20]	5.5%
Kessler et al (2020.12)	1	689	0.15	[0.00; 0.43]	0.3%
Shah et al (2020.7)	1	625	0.16	[0.00; 0.47]	0.3%
Menting et al (2020.1)	2	1185	0.17	[0.00; 0.40]	0.5%
Temkin et al (2020.10)	1	522	0.19	[0.00; 0.57]	0.2%
Wood et al (2020.8)	1	511	0.20	[0.00; 0.58]	0.2%
Singer et al (2020.10)	10	4751	0.21	[0.08; 0.34]	1.4%
Pavli et al (2020.9)	2	891	0.22	[0.00; 0.54]	0.3%
Petersen et al (2020.10)	88	36061	0.24	[0.19; 0.29]	6.3%
Zhang et al (2020.9)	21	8553	0.25	[0.14; 0.35]	2.1%
Scheier et al (2021.2)	8	2807	0.29	[0.09; 0.48]	0.6%
Kwon et al (2020.7)	6	2087	0.29	[0.06; 0.52]	0.5%
Deng et al (2020.10)	1	347	0.29	[0.00; 0.85]	0.1%
Maechler et al (2020.12)	14	4333	0.32	[0.15; 0.49]	0.9%
Aslam et al (2020.10)	42	11622	0.36	[0.25; 0.47]	1.9%
Olalla et al (2020.8)	2	498	0.40	[0.00; 0.96]	0.1%
LaCourse et al (2020.5)	1	230	0.43	[0.00; 1.29]	0.0%
Blumberg et al (2020.10)	6	1198	0.50	[0.10; 0.90]	0.2%
Santos et al (2020.12)	47	8037	0.58	[0.42; 0.75]	0.9%
Park et al (2020.4)	8	1143	0.70	[0.22; 1.18]	0.1%
Nishiura et al (2020.5)	4	565	0.71	[0.02; 1.40]	0.1%
van Buul et al (2020.12)	6	839	0.72	[0.14; 1.29]	0.1%
Marschner et al (2020.7)	1	139	0.72	[0.00; 2.12]	0.0%
Rashid−Abdi et al (2020.11)	1	131	0.76	[0.00; 2.25]	0.0%
Puckett et al (2020.12)	2	227	0.88	[0.00; 2.10]	0.0%
Lavezzo et al (2020.7)	29	2812	1.03	[0.66; 1.40]	0.2%
Akbarialiabad et al (2020.9)	19	1805	1.05	[0.58; 1.52]	0.1%
Goldfarb et al (2020.5)	9	757	1.19	[0.42; 1.96]	0.0%
Ibrahim et al (2020.8)	55	4617	1.19	[0.88; 1.50]	0.3%
Bosworth et al (2020.7)	16	1282	1.25	[0.64; 1.86]	0.1%
Tang et al (2020.7)	13	1027	1.27	[0.58; 1.95]	0.1%
Arnold et al (2021.1)	38	2882	1.32	[0.90; 1.73]	0.1%
Trahan et al (2020.11)	11	803	1.37	[0.57; 2.17]	0.0%
Cattelan et al (2020.8)	109	7595	1.44	[1.17; 1.70]	0.4%
Lombardi et al (2020.6)	28	1573	1.78	[1.13; 2.43]	0.1%
Massarotti et al (2020.8)	6	333	1.80	[0.37; 3.23]	0.0%
Cloutier et al (2020.8)	6	330	1.82	[0.38; 3.26]	0.0%
Hoxha et al (2020.7)	6244	280427	2.23	[2.17; 2.28]	5.8%
Roxby et al (2020.5)	2	80	2.50	[0.00; 5.92]	0.0%
Bender et al (2020.9)	8	318	2.52	[0.79; 4.24]	0.0%
Harada et al (2020.12)	33	1259	2.62	[1.74; 3.50]	0.0%
Mattar et al (2020.12)	18	686	2.62	[1.43; 3.82]	0.0%
Ly et al (2020.11)	46	1691	2.72	[1.94; 3.50]	0.0%
Hung et al (2020.9)	6	215	2.79	[0.59; 4.99]	0.0%
Grechukhina et al (2020.11)	44	1567	2.81	[1.99; 3.63]	0.0%
Ralli et al (2020.12)	9	298	3.02	[1.08; 4.96]	0.0%
Yousaf et al (2020.7)	6	195	3.08	[0.65; 5.50]	0.0%
Varnell et al (2021.1)	9	281	3.20	[1.14; 5.26]	0.0%
Abeysuriya et al (2020.9)	6	180	3.33	[0.71; 5.96]	0.0%
Olmos et al (2021.1)	14	413	3.39	[1.64; 5.14]	0.0%
Zhao et al (2020.8)	38	1060	3.58	[2.47; 4.70]	0.0%
Yau et al (2020.7)	12	330	3.64	[1.62; 5.66]	0.0%
Ochiai et al (2020.6)	2	52	3.85	[0.00; 9.07]	0.0%
Yassa et al (2020.7)	12	296	4.05	[1.81; 6.30]	0.0%
Al−Qahtani et al (2020.11)	116	2714	4.27	[3.51; 5.04]	0.0%
Lytras et al (2020.4)	35	783	4.47	[3.02; 5.92]	0.0%
Carroll et al (2020.10)	209	4586	4.56	[3.95; 5.16]	0.1%
Livingston et al (2020.10)	16	344	4.65	[2.43; 6.88]	0.0%
Marossy et al (2020.9)	115	2455	4.68	[3.85; 5.52]	0.0%
Figueiredo et al (2020.10)	9	184	4.89	[1.77; 8.01]	0.0%
Al−Shamsi et al (2020.11)	6	109	5.50	[1.22; 9.79]	0.0%
Martinez−Fierro et al (2020.10)	5	81	6.17	[0.93; 11.41]	0.0%
Sacco et al (2020.11)	12	179	6.70	[3.04; 10.37]	0.0%
Wadhwa et al (2020.12)	12	172	6.98	[3.17; 10.78]	0.0%
Gruskay et al (2020.6)	7	99	7.07	[2.02; 12.12]	0.0%
Blain et al (2020.7)	8	113	7.08	[2.35; 11.81]	0.0%
Rincón et al (2020.9)	14	192	7.29	[3.61; 10.97]	0.0%
Dora et al (2020.5)	18	235	7.66	[4.26; 11.06]	0.0%
Kennelly et al (2020.9)	290	2968	9.77	[8.70; 10.84]	0.0%
Bayle et al (2021.1)	24	241	9.96	[6.18; 13.74]	0.0%
Park et al (2020.12)	4	39	10.26	[0.73; 19.78]	0.0%
Milani et al (2020.6)	21	197	10.66	[6.35; 14.97]	0.0%
Yamahata et al (2020.5)	410	3711	11.05	[10.04; 12.06]	0.0%
Patel et al (2020.6)	14	126	11.11	[5.62; 16.60]	0.0%
Graham et al (2020.9)	54	464	11.64	[8.72; 14.56]	0.0%
Kirshblum et al (2020.7)	12	103	11.65	[5.45; 17.85]	0.0%
Blitz et al (2020.8)	45	382	11.78	[8.55; 15.01]	0.0%
Migueres et al (2020.9)	17	123	13.82	[7.72; 19.92]	0.0%
Abdelmoniem et al (2020.1)	29	203	14.29	[9.47; 19.10]	0.0%
Lan et al (2020.11)	16	104	15.38	[8.45; 22.32]	0.0%
Bianco et al (2020.5)	24	155	15.48	[9.79; 21.18]	0.0%
Corcorran et al (2020.8)	4	25	16.00	[1.63; 30.37]	0.0%
Shi et al (2020.10)	66	389	16.97	[13.24; 20.70]	0.0%
Kimball et al (2020.4)	13	76	17.11	[8.64; 25.57]	0.0%
Ladhani et al (2020.9)	97	518	18.73	[15.37; 22.09]	0.0%
Hcini et al (2020.2)	103	507	20.32	[16.81; 23.82]	0.0%
Tang et al (2020.11)	424	1970	21.52	[19.71; 23.34]	0.0%
Arons et al (2020.4)	27	76	35.53	[24.77; 46.29]	0.0%
**Random effects model**		.	**0.25**	**[0.23; 0.27]**	**100.0%**

Heterogeneity: I^2 99%, $\tau^2 < 0.0001$, χ^2_{94} 10374.27 (p 0)

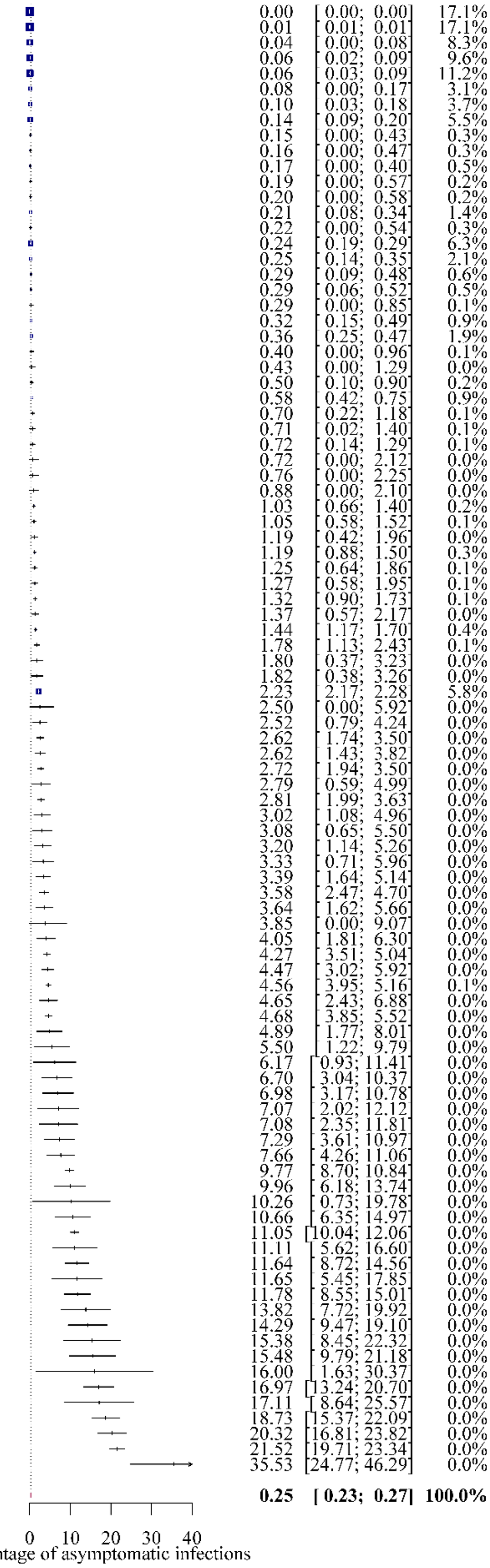

© 2021 Ma Q et al. *JAMA Network Open*.

eFigure 2. Percentage of Asymptomatic Cases Among the Confirmed Population

Study	Asymptomatic	Confirmed population	Percentage	95% CI	Weight
Deng et al (2020.10)	1	27	3.70	[0.00; 10.83]	1.4%
Maechler et al (2020.12)	14	333	4.20	[2.05; 6.36]	1.4%
Rashid−Abdi et al (2020.11)	1	21	4.76	[0.00; 13.87]	1.4%
LaCourse et al (2020.5)	1	13	7.69	[0.00; 22.18]	1.3%
Park et al (2020.4)	8	97	8.25	[2.77; 13.72]	1.4%
Zhang et al (2020.9)	21	235	8.94	[5.29; 12.58]	1.4%
Ibrahim et al (2020.8)	55	582	9.45	[7.07; 11.83]	1.4%
Tsou et al (2020.11)	10	100	10.00	[4.12; 15.88]	1.4%
Scheier et al (2021.2)	8	68	11.76	[4.11; 19.42]	1.4%
Zhang et al (2020.4)	3	25	12.00	[0.00; 24.74]	1.3%
Livingston et al (2020.10)	16	131	12.21	[6.61; 17.82]	1.4%
Yousaf et al (2020.7)	6	47	12.77	[3.23; 22.31]	1.4%
Park et al (2020.12)	4	30	13.33	[1.17; 25.50]	1.3%
Kwon et al (2020.7)	6	42	14.29	[3.70; 24.87]	1.3%
Martinez−Fierro et al (2020.10)	5	34	14.71	[2.80; 26.61]	1.3%
Blain et al (2020.7)	8	44	18.18	[6.79; 29.58]	1.3%
Menting et al (2020.1)	2	11	18.18	[0.00; 40.97]	1.2%
Al−Shamsi et al (2020.11)	6	32	18.75	[5.23; 32.27]	1.3%
Sacco et al (2020.11)	12	63	19.05	[9.35; 28.74]	1.4%
Lombardi et al (2020.6)	28	139	20.14	[13.48; 26.81]	1.4%
Ly et al (2020.11)	46	226	20.35	[15.10; 25.60]	1.4%
Akbarialiabad et al (2020.9)	19	86	22.09	[13.32; 30.86]	1.4%
Santos et al (2020.12)	47	211	22.27	[16.66; 27.89]	1.4%
Wi et al (2020.7)	25	111	22.52	[14.75; 30.29]	1.4%
Zhao et al (2020.8)	38	160	23.75	[17.16; 30.34]	1.4%
van Buul et al (2020.12)	6	25	24.00	[7.26; 40.74]	1.3%
Tang et al (2020.7)	13	52	25.00	[13.23; 36.77]	1.3%
Krüger et al (2021.1)	7	27	25.93	[9.40; 42.46]	1.3%
Kennelly et al (2020.9)	290	1105	26.24	[23.65; 28.84]	1.4%
Trahan et al (2020.11)	11	41	26.83	[13.27; 40.39]	1.3%
Cattelan et al (2020.8)	109	395	27.59	[23.19; 32.00]	1.4%
Bosworth et al (2020.7)	16	53	30.19	[17.83; 42.55]	1.3%
Nishiura et al (2020.5)	4	13	30.77	[5.68; 55.86]	1.2%
Grechukhina et al (2020.11)	44	141	31.21	[23.56; 38.85]	1.4%
Arnold et al (2021.1)	38	103	36.89	[27.57; 46.21]	1.4%
Varnell et al (2021.1)	9	24	37.50	[18.13; 56.87]	1.3%
Migueres et al (2020.9)	17	44	38.64	[24.25; 53.02]	1.3%
Rincón et al (2020.9)	14	36	38.89	[22.96; 54.81]	1.3%
Lavezzo et al (2020.7)	29	73	39.73	[28.50; 50.95]	1.3%
Corcorran et al (2020.8)	4	10	40.00	[9.64; 70.36]	1.1%
Patel et al (2020.6)	14	35	40.00	[23.77; 56.23]	1.3%
Pavli et al (2020.9)	2	5	40.00	[0.00; 82.94]	0.9%
Harada et al (2020.12)	33	79	41.77	[30.90; 52.65]	1.3%
Graham et al (2020.9)	54	129	41.86	[33.35; 50.37]	1.4%
Goldfarb et al (2020.5)	9	20	45.00	[23.20; 66.80]	1.2%
Shi et al (2020.10)	66	146	45.21	[37.13; 53.28]	1.4%
Mattar et al (2020.12)	18	35	51.43	[34.87; 67.99]	1.3%
Yassa et al (2020.7)	12	23	52.17	[31.76; 72.59]	1.2%
Yau et al (2020.7)	12	22	54.55	[33.74; 75.35]	1.2%
Singer et al (2020.10)	10	18	55.56	[32.60; 78.51]	1.2%
Arons et al (2020.4)	27	48	56.25	[42.22; 70.28]	1.3%
Ren et al (2021.2)	1749	3103	56.36	[54.62; 58.11]	1.4%
Tang et al (2020.11)	424	752	56.38	[52.84; 59.93]	1.4%
Kimball et al (2020.4)	13	23	56.52	[36.26; 76.78]	1.2%
Gruskay et al (2020.6)	7	12	58.33	[30.44; 86.23]	1.1%
Yamahata et al (2020.5)	410	696	58.91	[55.25; 62.56]	1.4%
Aslam et al (2020.10)	42	69	60.87	[49.35; 72.39]	1.3%
Ladhani et al (2020.9)	97	158	61.39	[53.80; 68.98]	1.4%
Al−Qahtani et al (2020.11)	116	188	61.70	[54.75; 68.65]	1.4%
Wadhwa et al (2020.12)	12	19	63.16	[41.47; 84.85]	1.2%
Blitz et al (2020.8)	45	71	63.38	[52.17; 74.59]	1.3%
Dora et al (2020.5)	18	27	66.67	[48.89; 84.45]	1.3%
Hung et al (2020.9)	6	9	66.67	[35.87; 97.46]	1.1%
Roxby et al (2020.5)	2	3	66.67	[13.32; 100.00]	0.8%
Carroll et al (2020.10)	209	310	67.42	[62.20; 72.64]	1.4%
Marossy et al (2020.9)	115	160	71.88	[64.91; 78.84]	1.4%
Ralli et al (2020.12)	9	12	75.00	[50.50; 99.50]	1.2%
Bayle et al (2021.1)	24	32	75.00	[60.00; 90.00]	1.3%
Hoxha et al (2020.7)	6244	8325	75.00	[74.07; 75.93]	1.4%
Heini et al (2020.2)	103	137	75.18	[67.95; 82.42]	1.4%
Lan et al (2020.11)	16	21	76.19	[57.97; 94.41]	1.3%
Petersen et al (2020.10)	88	115	76.52	[68.77; 84.27]	1.4%
Figueiredo et al (2020.10)	9	11	81.82	[59.03; 100.00]	1.2%
Abeysuriya et al (2020.9)	6	7	85.71	[59.79; 100.00]	1.2%
Blumberg et al (2020.10)	6	7	85.71	[59.79; 100.00]	1.2%
Massarotti et al (2020.8)	6	7	85.71	[59.79; 100.00]	1.2%
Lytras et al (2020.4)	35	40	87.50	[77.25; 97.75]	1.3%
**Random effects model**		.	**40.50**	**[33.50; 47.50]**	**100.0%**

Heterogeneity: $I^2 = 99\%$, $\tau^2 = 0.0919$, $\chi^2_{79} = 8735.46$ ($p = 0$)

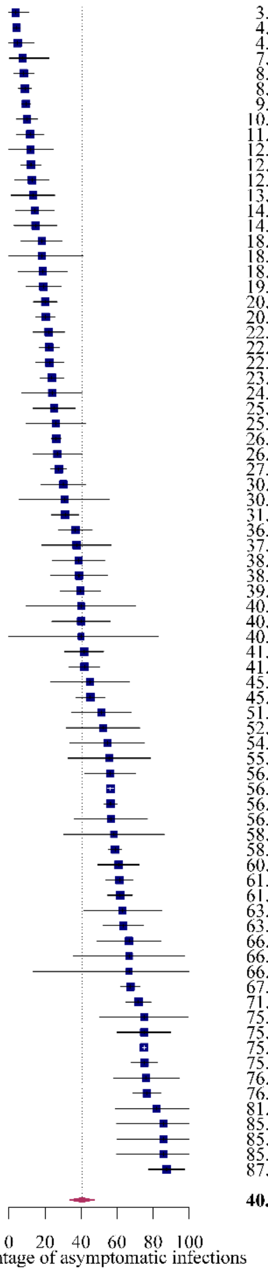

© 2021 Ma Q et al. *JAMA Network Open*.

eFigure 3. Percentage of Asymptomatic Cases Among the Tested Population by Subgroups, Using the Knapp-Hartung Adjustments

Subgroup	No. of studies	I-squared	P value	Percentage (%(95%CI))	Weight (%)
**Location**					
Africa	1	-	-	14.29 (9.47-19.10)	0
Asia	25	98%	<0.01	0.05 (0.00-0.15)	79.3
Europe	35	99%	<0.001	0.90 (0.53-1.27)	17.3
North America	32	97%	<0.01	0.47 (0.03-0.90)	3.4
South America	2	0%	0.48	2.87 (0.00-7.41)	0
**Development level**					
Developed	74	99%	<0.001	0.70 (0.46-0.95)	27.0
Developing	21	98%	<0.01	0.04 (0.00-0.12)	73.0
**Study population**					
Air / Cruise travel	8	99%	<0.01	2.02 (0.00-4.94)	8.6
Close contact	6	98%	<0.01	1.46 (0.00-3.65)	6.4
Community residents	14	95%	<0.01	0.40 (0.00-0.96)	22.3
Healthcare workers / Inhospital patients	37	92%	<0.01	0.75 (0.39-1.11)	49.0
Nursing home residents / staff	17	98%	<0.01	4.52 (1.87-7.17)	8.0
Pregnant women	13	94%	<0.01	2.34 (0.44-4.23)	5.7
**Publication period**					
June and before	21	98%	<0.01	0.09 (0.00-0.34)	20.3
July and after	74	99%	<0.001	0.29 (0.13-0.46)	79.7
**Sample size**					
1-100	8	84%	<0.01	6.74 (0.63-12.86)	0
100-1000	49	94%	<0.01	0.69 (0.19-1.18)	1.8
1000-10000	30	99%	<0.001	0.27 (0.00-0.56)	23.4
10000-	8	100%	<0.001	0.23 (0.00-0.75)	74.8
**Study design**					
Case series	7	92%	<0.01	0.55 (0.00-1.55)	10.1
Case series on transmission	3	7%	0.34	0.18 (0.00-0.50)	5.4
Cohort studies	41	98%	<0.001	2.98 (1.63-4.34)	34.1
Cross-sectional studies	44	99%	<0.001	1.55 (0.73-2.38)	50.4
**Study quality**					
Low	53	95%	<0.01	0.22 (0.07-0.37)	21.6
Moderate	25	100%	<0.001	0.64 (0.22-1.06)	24.5
High	17	99%	<0.01	0.05 (0.00-0.21)	54.0
**Male: female ratio**					
0-0.5	23	94%	<0.01	2.71 (1.12-4.31)	48.5
0.5-1.0	18	98%	<0.01	3.91 (0.77-7.05)	32.7
1.0-1.5	6	96%	<0.01	2.26 (0.00-7.21)	10.8
1.5-	4	89%	<0.01	1.40 (0.00-5.06)	7.9
**Average age**					
<20	2	84%	0.01	1.27 (0.00-16.74)	7.8
20-39	12	94%	<0.01	2.42 (0.21-4.62)	30.4
40-59	7	92%	<0.01	0.84 (0.00-2.40)	31.5
60-	10	99%	<0.01	3.69 (0.00-8.40)	30.3
Combined percentage	95	99%	<0.001	0.25 (0.11-0.39)	100.0

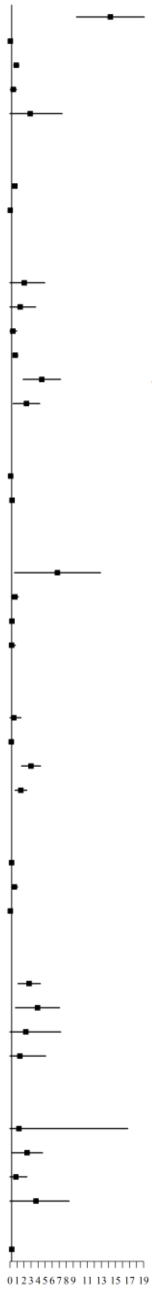

© 2021 Ma Q et al. *JAMA Network Open*.

eFigure 4. Percentage of Asymptomatic Cases Among the Confirmed Population by Subgroups, Using the Knapp-Hartung Adjustments

Subgroup	No. of studies	I-squared	P value	Percentage (%(95%CI))	Weight (%)
**Location**					
Asia	20	99%	<0.001	27.58 (18.05-37.12)	26.6
Europe	31	99%	<0.001	44.18 (34.03-54.33)	40.6
North America	25	90%	<0.01	46.32 (37.91-54.73)	31.5
South America	1	-	-	51.43 (0.00-100.00)	1.3
**Development level**					
Developed	62	99%	<0.001	43.51 (37.31-49.72)	80.1
Developing	15	99%	<0.01	28.46 (16.46-40.47)	19.9
**Study population**					
Air / Cruise travel	8	92%	<0.01	52.91 (33.21-72.61)	9.8
Close contact	6	98%	<0.01	26.94 (0.00-54.33)	8.2
Community residents	9	98%	<0.01	39.74 (15.63-63.86)	12.0
Healthcare workers / Inhospital patients	27	91%	<0.01	30.01 (22.67-37.34)	35.3
Nursing home residents / staff	17	99%	<0.001	47.53 (36.89-58.17)	22.3
Pregnant women	10	94%	<0.01	54.11 (34.52-73.70)	12.4
**Publication period**					
June and before	16	97%	<0.01	43.68 (29.96-57.41)	20.1
July and after	61	99%	<0.001	39.72 (33.49-45.96)	79.9
**Sample size**					
1-100	51	92%	<0.01	40.86 (33.82-47.91)	63.7
100-500	20	99%	<0.01	36.98 (25.73-48.22)	27.8
500-	6	100%	<0.001	47.06 (21.59-72.53)	8.5
**Study design**					
Case series	6	87%	<0.01	28.52 (3.65-53.38)	7.8
Case series on transmission	3	45%	0.16	15.85 (0.00-59.69)	3.6
Cohort studies	38	98%	<0.01	40.96 (33.47-48.46)	49.2
Cross-sectional studies	30	99%	<0.001	44.47 (34.70-54.24)	39.4
**Study quality**					
Low	40	95%	<0.01	38.93 (30.70-47.16)	50.9
Moderate	21	99%	<0.01	48.06 (38.01-58.11)	28.1
High	16	99%	<0.001	32.94 (20.67-45.20)	20.9
**Male: female ratio**					
0-0.5	21	96%	<0.01	41.27 (28.15-54.38)	50.8
0.5-1.0	12	98%	<0.01	31.29 (18.14-44.44)	30.4
1.0-1.5	4	97%	<0.01	55.09 (0.00-100.00)	9.3
1.5-	4	90%	<0.01	39.98 (18.50-61.46)	9.6
**Average age**					
<20	2	88%	<0.01	60.21 (0.00-100.00)	7.5
20-39	9	98%	<0.01	49.49 (26.65-72.33)	37.3
40-59	5	88%	<0.01	32.48 (3.57-61.39)	22.0
60-	8	97%	<0.01	33.83 (19.09-48.56)	33.1
Combined percentage	77	99%	<0.001	40.50 (34.94-46.07)	100.0

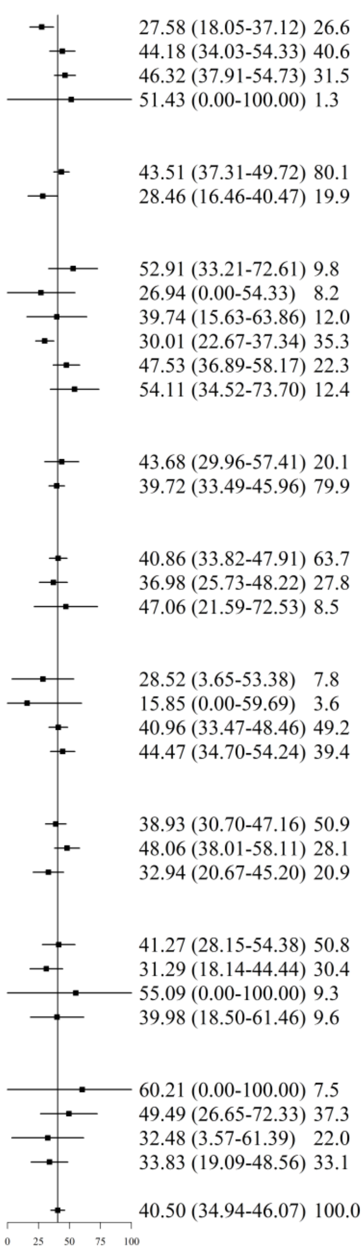

© 2021 Ma Q et al. *JAMA Network Open*.
